# A qualitative study of the implementation and organization of the national Greenlandic addiction treatment service

**DOI:** 10.3389/frhs.2024.1219787

**Published:** 2024-03-06

**Authors:** Julie Flyger, Christina Viskum Lytken Larsen, Else Jensen, Birgit Niclasen, Anette Søgaard Nielsen

**Affiliations:** ^1^ Unit of Clinical Alcohol Research, Clinical Institute, University of Southern Denmark, Odense, Denmark; ^2^ Institute of Nursing and Health Science, Ilisimatusarfik—University of Greenland, Nuuk, Greenland; ^3^ Center for Public Health in Greenland, Institute of Public Health, University of Southern Denmark, Copenhagen, Denmark

**Keywords:** implementation, alcohol, cannabis, addiction, treatment

## Abstract

**Background:**

Alcohol and cannabis use constitutes the major public health problems in Greenland. Thus, it is important to assess if Allorfik, a new national outpatient addiction treatment service introduced in 2016, was implemented successfully and how it is perceived. Allorfik introduced local treatment centers offering a treatment methodology (motivational interviewing and cognitive therapy) new to addiction treatment in Greenland with limited evidence from Indigenous populations such as the Greenlandic. The present study investigates the implementation of Allorfik from the perspective of those engaged in the process and the field.

**Methods:**

Data consisted of transcribed interviews with 23 individuals from both Allorfik and organizations collaborating with or supposed to collaborate with Allorfik. The theme of the interviews was their perspectives on the implementation process, enablers, and obstacles in the process and how Allorfik was performing at the time of the interview. The interview guide was informed by implementation theory. The transcribed material was analyzed using a general inductive approach.

**Results:**

The analysis resulted in three overall and interconnected themes, namely, implementation, collaborations, and challenges. The implementation was overall considered a success by the interviewees as all components were implemented as planned with a few adaptions, e.g., a treatment guideline update. The collaborations are considered challenging but important to all interviewees. Collaborations seem to rely on personal commitment as opposed to well-defined structures, making it unstable and vulnerable to changes in staff. One of the main challenges highlighted by the interviewees is the number of problems other than addiction among people in treatment, which makes addiction treatment and recovery difficult to achieve. Nevertheless, the high levels of other problems being treated in Allorfik highlights the need for easily accessible therapy as many find that Allorfik is the only place to turn to in times of crisis.

**Conclusion:**

Allorfik seems to have been implemented in accordance with original intentions and plans for addiction treatment service but has also become more than just a service for addiction treatment with easy access in a country with vast distances and limited resources.

## 1 Background

Alcohol and cannabis use has been one of the biggest health and social challenges in Greenland (1, 2); thus, addiction treatment services are important for a portion of the population. In 2013, Naalakkersuisut (the Government) was imposed by Inatsisartut (the Parliament) to (1) analyze the local need for treatment for addiction and the societal gains in providing the treatment free of charge (3) and (2) to present a plan for Inatsisartut for the future national addiction treatment service in spring 2015 (4). The plan presented was approved and funded through the Finance Act starting in 2016. The central aim of the new national plan was to provide differentiated treatment methods and treatment as close as possible to the homes of the people in need. Alongside, a privately established facility using the 12-Step Model for treatment in the capital, Nuuk, the government thus established a new treatment system, Allorfik, for outpatient treatment of addiction to alcohol, cannabis, and gambling, free of charge for users (4). The plan described that the foundation for treatment methods should be motivational interviewing (MI) and cognitive behavioral therapy; however, access to the 12-Step Model (5) for treatment with approved counselors should be continued. The recommendation of methods of the plan was made with respect to being sensitive to the Greenlandic context; however, with scarce and not easy-to-convert evidence on addiction treatment methods for Inuit or Indigenous people, the recommendation of treatment methods was based on the available medical evidence and best practices from Western societies.

In general, evidence on methods in addiction treatment among Inuit and Indigenous people is sparse; however, a review paper from Andersen et al. examined the treatment for alcohol addiction among Indigenous people (6). This review identified 19 studies, primarily from North America, and tentatively concluded that medical treatment with naltrexone seemed to be effective in reducing heavy drinking, and MI seemed to be an effective psychosocial treatment approach among the Indigenous populations studied. Another important point from Andersen et al. was that although the basis of the review was scarce and the studies included were hard to compare, community-driven approaches, traditional healing methods, and inclusion of other cultural elements seem to be very important in providing good treatment for alcohol use disorders across different Indigenous populations (ibid). A few other examples supporting the use of MI in interventions for people with alcohol addiction in Indigenous populations were found (7, 8). MI (9) and cognitive behavioral therapy (10) were well-established and widely used in other countries, e.g., England (11) and Denmark (12). However, these treatment methods were unfamiliar to addiction treatment in Greenland before the establishment of Allorfik, and in the case of the 12-Step Model for treatment, the efficacy of the methods has primarily been evaluated in Western populations and not among Inuit.

Implementation of a treatment strategy involving both new methods and a new organization nationwide in Greenland can be considered a huge task, which is a type of task that has not been studied before. Studies on the implementation of addiction treatment so far have primarily focused on, e.g., barriers toward the implementation of evidence-based treatment (13) and the implementation of new elements in the established treatment services, e.g., telemedicine (14) or services for an underserved target group (15); however, studies on the implementation of new addiction treatment services and implementation of new methods nationwide to our knowledge have not been performed yet. Furthermore, the treatment service in Greenland was particularly interesting because of the lack of evidence-based knowledge about treatment in Indigenous populations in general, because the methods of MI and cognitive behavioral therapy specifically were new to addiction treatment in Greenland when implemented, and because of the high impact of addiction on public health and to human suffering in Greenland (1, 2, 4, 16). So far, only a few studies have been published about the functioning and outcome of treatment delivered by Allorfik (17–20). These studies primarily investigated specific aspects of the treatment and the characteristics of people attending treatment, not of the provision and the organization of the treatment service itself. Thus, the study “Evaluation of the implementation of best practice models in the treatment of alcohol and other addictions in Greenland” was initiated, which aimed to evaluate the implementation of Allorfik from a series of angles. The present study was the first sub-study of this overall evaluation, and the following sub-studies will investigate the quality of treatment and how the people in treatment manage after treatment.

The present paper aimed to investigate the implementation process and organization of the new treatment service, Allorfik in Greenland seen through the eyes of those involved, i.e., planners, staff, and collaborators. The objective was to investigate the barriers and facilitators of the implementation process and assess if Allorfik was implemented in adherence with the original plans for a new addiction treatment service according to those involved in the process.

## 2 Materials and methods

### 2.1 Setting

Greenland is the home of approximately 56,600 inhabitants with the majority living in the capital municipality, Kommuneqarfik Sermersooq. Greenland was a Danish colony until 1953 and is now part of the Kingdom of Denmark with a self-rule status since 2009. Approximately 60% of the population live in one of the five largest towns (with an Allorfik center), 25% live in smaller cities, and ∼15% live in settlements. Almost 90% of the population are Inuit or Inuit descendants, and the largest minority is Danish (21). The infrastructure in Greenland is difficult since no roads connect towns or settlements, and transportations are thus done by air or sea. The public administration is similar to many of the Scandinavian countries, e.g., all health services are free of charge (22).

During a period of 3 years, Allorfik stepwise opened, from South to North Greenland, outpatient treatment centers in the largest town in each of the five municipalities. As of the summer of 2018, Allorfik has established a treatment center in each of the five main cities: Qaqortoq, Nuuk, Sisimut, Aasiaat, and Ilulissat. The implementation of Allorfik centers started in Qaqortoq in November 2016 and ended in July 2018 in Aasiaat. People in need of addiction treatment can be referred to treatment by a social worker or employer or simply seek treatment themselves by contacting their local Allorfik center, free of charge. In addition, people not living in a town with an Allorfik center have an additional series of treatment options via the Internet or telephone or local treatment by a traveling private partner using the 12-Step Model for treatment in smaller towns. Moreover, people living in settlements and towns without an Allorfik treatment center can be temporarily relocated for an intensive day treatment in Nuuk, again with a private partner using the 12-Step Model of treatment method. The 12-Step Model of treatment method in Nuuk implies an intensive daily recovery process oriented towards total abstinence and accepting addiction as a chronic disease, and the counselors’ training builds upon the 12-Step Model of treatment (23), the Alcohol Anonymous movement (24) in Greenland, and the Minnesota Model for treatment (5).

After treatment conclusion, a 6-month group-based aftercare program is available for people who have been in treatment, irrespective of the treatment method during treatment (i.e., an offer to both people receiving treatment in Allorfik and for people receiving the 12-step treatment). In 2021 almost 1,000 people were referred to treatment, and 741 treatment courses were completed (25), but the total number of people referred to treatment and completed treatment courses dropped to 753 and 543, respectively, in 2022 (26). The decline in numbers in 2022 was caused by several factors including COVID-19 and a massive water damage to the treatment center in Nuuk, which caused the center to temporarily close and later move its facilities.

From 2018, Allorfik consisted of five treatment centers, a central administration and knowledge center located in Nuuk, and, in addition through a partnership agreement with a private provider, referred persons in need to intensive day treatment in Nuuk. In the local treatment centers, there was a minimum of three counselors available, offering treatment courses. The internal organization of Allorfik has changed a bit throughout the years in Allorfik. In the early days, a cognitive behavioral psychotherapist and, later, two psychologists were employed to support the implementation process. The psychotherapist and the psychologists' main function was to be part of the management group and support the Allorfik centers and the counselors individually, to train new employees, help implement guidelines, and supervise the counselors both in groups and individually. However, after a few years, these positions were no longer staffed, and the supervision and training were instead handled either from within the organization or by experienced external consultants, both from Greenland, by means of visiting supervisors from Denmark and via online support.

### 2.2 Methodology

The present study's focus on the establishment and implementation process of the treatment service has informed both the study design and analysis. While the implementation of Allorfik had taken place prior to the start of the present study, the focus of the present study was to understand what had influenced the implementation process, seen in retrospect, and not a process evaluation taking place alongside the implementation. According to Per Nilsen's terminology (27), the study used the methodological framework RE-AIM (28) as inspiration for forming this study—especially when developing the interview guide and deciding who to invite for interviews. RE-AIM has five main constructs: reach, effectiveness, adoption, implementation, and maintenance. A central assumption of how the impact of an intervention relies on the combined effect of the five constructs (29). The interview guide was developed around the RE-AIM framework constructs and the descriptions of Allorfik to ensure the inclusion of all elements of implementation and the central treatment elements of Allorfik. The interview guide was discussed with both the author group and the reference group and adjusted accordingly.

This study has been approved by the Research Ethics Committee for Scientific Health Research in Greenland.

### 2.3 Reference group

Inspired by community-based participatory research (CBPR) and the previous work in Greenland with community engagement and strength-based research (30–32), the overall study established a reference group. It was not the focus of either the present sub-study or the overall study to have a complete CBPR approach but with inspiration from CBPR to do a study with emphasis on being inclusive and respectful to the community perspectives to research project and to have a transparent process with a strong link to the context of the study. Therefore, the study established a reference group to advise on the project; discuss the research plans, process, and results; and disseminate the results and recommendations, if the study found any. The participants of the reference group were chosen to represent different geographical areas in Greenland and different professions, consisting of five people in total: one from health research in Greenland, two Allorfik staff (not in a managing position), one from the central administration, and one collaborating partner from a municipality. During the first meeting, the overall research plan and the interview guide for this study were discussed. During the second meeting, the participants were presented with the tentative findings of the present study, i.e., quotes from the interviews and preliminary identification of emerging themes to let their perspectives inform the process, analysis, and conclusions of this study. None of the participants in the reference group were reimbursed for their time, and participation was voluntary. Meetings were held within normal working hours and lasted around an hour each.

### 2.4 Data

**Figure 1:**
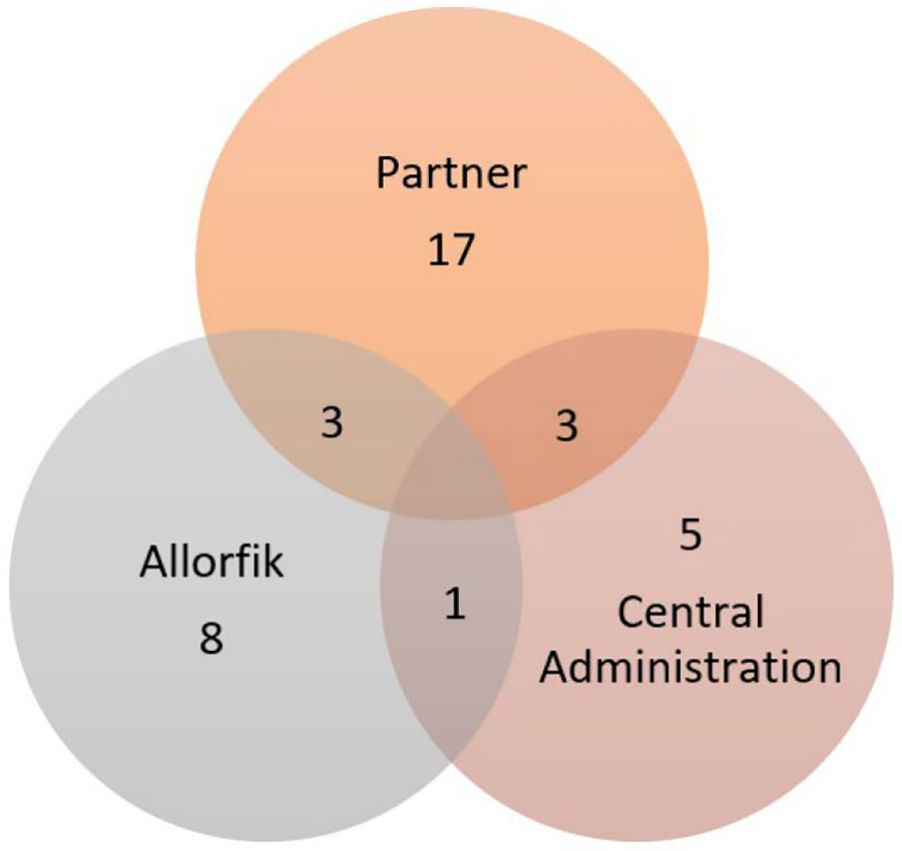
The interviewees’ place of employment. Each circle presents the total number of interviewees who had experience from employment with a partner organization, Allorfik, and central administration and those who had experience from more than one place with the overlaps.

The data for the present study consisted of semi-structured interviews with 23 individuals. The interviewees were recruited based on their current or former employment and represented internal Allorfik staff, government officials, and personnel from collaborating partner organizations, e.g., the healthcare system, municipalities, and local NGOs. The interviewees were recruited based on their function in work, and of the 23 interviewees, there is an equal part of people in managing and in employee positions. The interviewees from Allorfik were counselors, counselor group leaders, training and supervision staff, and the director. The interviewees from the municipalities were social workers, department managers, local prevention workers, family center workers, and employment agents who had or could have collaborated with Allorfik. The interviewees from the healthcare system were department leaders, midwives, healthcare workers, and collaborators or potential collaborators with Allorfik. Most people interviewed were geographically based centrally in Nuuk, five were from North Greenland, three were from South Greenland, three were from Middle Greenland, and one person was from East Greenland. Recruitment of interviewees ended as a good coverage of both geographical areas, organizational perspectives were included, and no new perspectives emerged in the interviews. Supplementary Appendix I and Figure 1 provide an overview of the interviewee's places of employment and time of involvement as either a member of staff or collaborator. No people living in settlements were included in interviews as the access to treatment there has not changed substantially with the implementation of Allorfik. Due to the diversity of geographical locations, time of involvement, and the employment positions of the interviewees, the interviews were carried out individually.

During the interviews, the conversations concentrated on the interval of time the individual was involved in or collaborated with Allorfik. A few people had been involved throughout the whole process from initiation until the present day, some had been involved only in the early days of establishing Allorfik, and some were relatively new in their involvement or relation to Allorfik as illustrated in Table 1 in the Supplementary Appendix I. When applicable, the topics of the conversation were the establishment, difficulties, and strengths in the implementation process and how the treatment service was working at the time of the interview. The interviewees were also asked for their perspectives on the suitability of the implemented treatment strategy in this population. The first interview was conducted in January 2021, and the last interview was in October 2021. Due to COVID-19, all interviews were collected over a long period of time through video calls via Zoom or Teams and recorded as video conversations. However, it is only the recorded audio from these conversations that is used for the analysis. The shortest interview lasted approximately 30 min and the longest one and a half hours—most of them lasted ∼45–50 min. All interviewees were given the option of performing the interview in either Greenlandic or Danish. One person preferred Greenlandic and was interviewed by a local interviewer, Else Jensen, who also transcribed and translated the interview to Danish for inclusion in the analysis. All other interviews were conducted by first author, Julie Flyger, and transcriptions of the interviews were conducted by Julie Flyger and student assistant, Camilla Dahl Olsen. Julie Flyger has a background in public health and was a young researcher with previous experience and training in conducting interviews. The interview process was supervised by authors Anette Søegaard Nielsen and Christina V. L. Larsen who have extensive experience in conducting qualitative research projects. None of the participants were reimbursed for their time, and participation was voluntary. The meetings were held within normal working hours at a time of their choosing, and all provided informed consent prior to the interview (Supplementary Appendix II).

### 2.5 Data analysis

Each interview was transcribed verbatim. The data from the interviews were condensed and analyzed using the general inductive approach by Thomas (33). Each transcript was carefully read through, and all meaningful phrases were coded. After the initial coding, all codes were read again looking for patterns, meanings, and common denominators, and after yet another read-through, they were finally organized into themes. All transcriptions and coding were done using NVivo software. The process was a simple thematic analysis inspired by the description of Braun and Clarke (34). The transcribed interviews were read through by the same researcher who conducted the interviews and shared with authors Anette Søegaard Nielsen and Christina V. L. Larsen. The preliminary themes and quotes were discussed with the reference group as described and discussed in the author group too for the final analysis to be established. In the author group, there were both members of staff and stakeholders to validate the findings, but they were not involved in the process of analysis. The analysis was performed in Danish languages and only the quotes presented in the Results section were translated into English.

## 3 Results

Several interviewees had changed job positions since the initial implementation of Allorfik. Figure 1 provides an overview of the interviewee's employment places during the implementation period and the overlap in experiences. As can be seen, several interviewees had experiences from several places and job positions. Few Allorfik staff have been involved throughout the whole period of implementation. Table 1 in Supplementary Appendix I provides an overview of which period each interviewee presented and talked about. With Greenland having such a small population, it would not be possible to secure the interviewees’ anonymity if we referred to the interviewee by means of their job title and place of living, and although all have consented to being quoted, their anonymity was prioritized in accordance with the Science Ethics Committees guidance of good research practices in Greenland. Furthermore, frequent job changes were not abnormal in the Greenlandic society, and the changes in employment some interviewees have had provided different perspectives of Allorfik depending on the period of implementation discussed. In the present paper, when referring to the interviewees, it was thus important to demonstrate if the quote came from the perspective of a member of staff (in this case defined as either being from central administration or an Allorfik employee) or a collaborator (defined as staff from the municipality, healthcare system, or NGO or potential collaborators with Allorfik) as these two angles provide different perspectives on the implementation process and organization.

### 3.1 Themes

**Figure 2:**
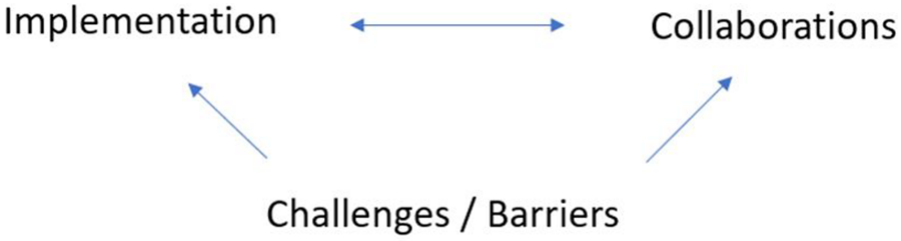
The themes and their cohesion.

The thematic analysis identified three major themes in the data: the implementation, collaborations, and challenges/barriers and a series of sub-themes. The themes will be elaborated on in the following sections and are very much interlinked as demonstrated in Figure 2.

### 3.2 Implementation

Many interviewees reported that they found the implementation of Allorfik to be difficult, which to some extent still was. One of the most emphasized encounters in the implementation was the recruitment and retention of staff in Allorfik. One interviewee described a struggle at the beginning of implementation, which was the recruitment of staff who were trained in or had experiences from another treatment tradition and methodology than what was supposed to be implemented:

I think that has been one of the biggest barriers, it is simply that you have had many of them from the 12-step treatment and it's known that, if you must learn something new and unlearn the old, it is very difficult. (5, collaborator experience)

On top of this, several of the interviewees from Allorfik described how the working conditions were difficult. With the vast distances and difficult infrastructure in the country, some staff described a feeling of being alone and very far away from management, and one described how she had not participated in an annual appraisal and improvement interview in several years. Nevertheless, many of the interviewed staff members also highlighted that Allorfik was an improvement to the previous service and were genuinely happy about their work there:

From the bottom of my heart, it has made me happy that it was the difference I have helped to make. (12, staff experience)

Several staff members expressed how a continuous focus within Allorfik on the quality of treatment resulted in a revised treatment guideline^1^ implemented in June 2020, and this revision improved the treatment sessions for both them and the persons in treatment. This change also increased the staff’s feeling of job satisfaction. Moreover, the implementation of the improved treatment guideline also seemed to be reflected in an increased quality of the treatment, also noticed by collaborators:

They document their methods and, and use their methods in the records and with the patients, and they are especially good at the motivational interview, so I think it has increased quite significantly, I would say, compared to the first years, which were the definitely not good. So, in that way, I think it, it has been a very big improvement in quality here… so I think they are delivering what they must. But they don't deliver it at a high level because there has been this turnover, so you start over with new staff and so on. But those who are now there and have remained there, they deliver at a level that completely corresponds to the Danish level. (5, collaborator experience)

#### 3.2.1 Management

A key factor in the implementation process seemed to be the stability of the management of Allorfik throughout the years. Several interviewees mentioned how a clear plan for implementation laid out by unchanging management probably was one of the reasons why the implementation of Allorfik had been relatively smooth, quick, and secure:

She had it all designed and if it had been another manager who had started, it would have taken much longer time to build it up. No doubt about it. (18, staff experience)

However, a strong management during the process, concentrated in one central person, situated in the capital also created a risk for lack of communication and decisions being taken that did not consider local aspects, making it difficult for staff and partners to navigate. Some of the informants directly expressed how management had stepped on the toes of some people during the process and missed collaboration opportunities. Nevertheless, the overall impression was that management was given credit for being receptive to potential allies in the implementation process and for having performed a tough job with the changes in staff and also being responsible for many tasks external to Allorfik, e.g., training courses and education of counselors. One person highlighted the difficulties being a leader of addiction treatment services must have involved when most of the staff themselves or a close relative of theirs have had problems with addiction and other problems, due to the high burden of addiction and social problems in Greenland:

… they also have some problems themselves. Psychological, psychological problems and so on, which characterize a lot of the staff…. (5, collaborator experience)

#### 3.2.2 Language

Albeit not being part of the original plans but emerging during the process of implementation was the need for establishing a common treatment “language” or vocabulary within Allorfik: a need that was important for counselors:

It is more than 98% of our,… um…, of those who come for treatment with us, are primarily Greenlandic speaking and that means that it is incredibly important that… um… that they are met not only linguistically but also culturally… and find out, … um…, what is it that what we need in terms of methods. How do we talk about things? What are some expressions we use? … or what kind of words are we giving a special meaning here? Which [words] covers some of the stuff that we'd like to have the opportunity to talk to people about…. (17, staff experience)

### 3.3 Collaborations

When the interviews turned to the subject of collaboration between Allorfik and the rest of the Greenlandic society, a series of perspectives emerged, of which three were dominant: the healthcare system, the municipalities as collaborating organizations, and the outreach work done by Allorfik.

#### 3.3.1 The healthcare system

The interviewees from the healthcare system all perceived the collaboration with Allorfik as good, albeit not very close. From the point of view of healthcare professionals, there was not much to collaborate on. One person with a healthcare management position reflected on how the healthcare services have not been great at facilitating collaboration, whereas others highlighted the limited resources in the healthcare systems as an important barrier to do more collaboration. Several informants from the healthcare system would like to be able to refer people from the healthcare system directly to treatment Allorfik to avoid non-appearances or needing to involve the municipalities:

So, I don't know if you can somehow work for some kind of referral system. (10, collaborator experience)

A common argument for wishing for a direct referral system and not involving the municipalities was that some treatment seekers and even employees in the healthcare system found that approaching the municipalities indicated a need for social services rather than for addiction treatment:

Many of our employees who actually need a, an, an addiction treatment course, well they don't, they don't really need to come down to their case manager, because they have an income and they have a home and, and things like that but, but they do need to get in to addiction treatment service, because otherwise they lose their job. (1, both staff and collaborator experience)

#### 3.3.2 The municipalities

The informants from the municipalities illustrated how the collaboration with Allorfik varied across the country. The collaborations varied both between different municipalities and Allorfik and within the different municipality units in a single city and the local Allorfik center. The collaboration was perceived as mutual and satisfactory in some places, and the collaboration was described as sparse or non-existent in other areas:

Yes, it was very general, the whole time it has been very superficial I, I have maybe been to three meetings up there in over five years. (10, collaborator experience)

The differences indicated that formal structures for collaborations were not established throughout the country:

We can’t really find out, or something like that, how do we cooperate and what do the people who come to Allorfik do, uhm, who refers, how do we refer and various things like that. (3, collaborator experience)

Overall, the collaboration with municipalities seemed to rely on the attitudes of and relations between specific individuals in both Allorfik and the municipality rather than being embedded in formal structures. Thus, when there was a change of staff in either organization, the collaboration often needed to be rebuilt or simply collapsed. However, some informants described the collaboration between Allorfik and social services as running very smoothly in a few specific areas, namely, when there were children involved and to some extent between Allorfik and a municipality's health prevention worker^2^. However, the collaboration between Allorfik and other sectors and institutions was overall described as varying and fluctuating, illustrated by an informant explaining the collaborations varied even within a single city:

Well, my impression is that they have a good collaboration, for example with the hospital. The collaboration with the Family services that, that is also something that must be constantly improved. (3, collaborator experience)

With some of the local Greenlandic NGOs, there does not seem to be any type of collaboration Allorfik, while with others there was a formal agreement and a good relationship. With some collaborators the beginning was difficult but with time the collaboration has improved significantly.

#### 3.3.3 Outreach work

Several informants from the staff, collaborators such as the municipalities, healthcare system, and the NGOs expressed that it was of great importance when employees from Allorfik performed outreach visits to other facilities, meeting both staff and citizens—potential collaborating partners and people in need of treatment:

But, but we are really happy about the cooperation we have around their counselors, who come here and give presentations for the patients and also uh presentations for our staff uh, so that they are also better equipped to, to talk or deal with um this. (1, both staff and collaborator experience)

In continuation, one person from Allorfik described how the opportunities for collaboration were great but depended very much on the person's interests:

So, if you want to or if you have the energy for it, there are plenty of opportunities for collaboration. (6, both staff and collaborator experience)

### 3.4 Challenges and barriers

**Figure 3:**
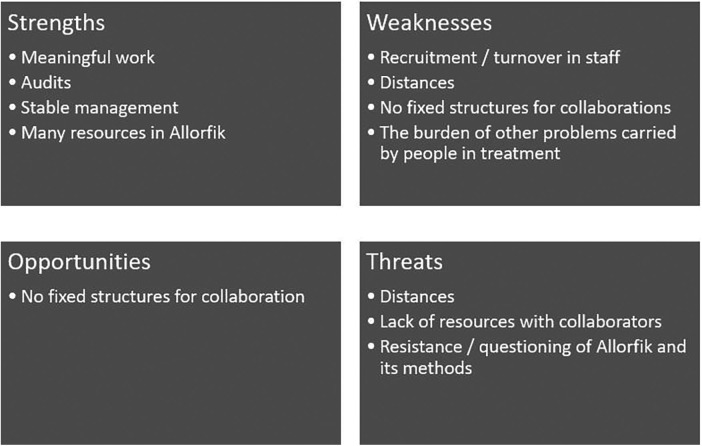
Strengths, weaknesses, opportunities, and threats.

When the interviewees were asked if they could identify any difficulties for Allorfik—both past and present—a series of angles were highlighted. These challenges have been used in Figure 3 where these have been allocated to the four groups: strengths, weaknesses, opportunities, and threats.

#### 3.4.1 Within Allorfik

One shared experience by the staff of Allorfik was the challenges within the organization itself. At the time of establishment, the challenges were primarily practical and related to the uncertainty of what the new treatment strategy contained: who to hire, where to situate the treatment facilities, and what kind of interior was needed, in addition to unforeseen events such as water damage to a facility. A persistent challenge was, however, the constant staff turnover, which was linked to both the changes in treatment methodology (from the 12-Step Model to MI and CBT) and to contractual restrictions at the government level making it difficult to attract and retain staff:

They have struggled with the huge, huge turnover. (16, staff experience)

Another challenge described by the informants was the difficulties in promoting the success stories from Allorfik, in contrast to the more broadly known narratives of successful 12-step addiction treatment courses, told by influential members of the Greenlandic society about the old treatment system. In some of the interviewees' experiences, this narrative was considered to have heightened some of the preferences of people in treatment for specific treatment methods rather than others. Since some of the leading figures in the Greenlandic society had experiences from the old treatment system in addition to them being involved in politics, some interviewees indicated that this might have put political pressure on Allorfik to question the organization's general way of working and choice of treatment methods.

#### 3.4.2 The people in treatment

The informants described massive levels of problems and challenges among the people seeking help with Allorfik and how these multi-problems impacted the implementation process since they might be hard for the staff to handle. One interviewee pointed to these multi-problems as a driver for the addiction problems:

After all, that is what makes them fall or they become addicts, too, because they have something in their baggage that they have to process. And I think it means a lot, if you want to completely recover from your addiction, that you also, uh, process those things at the same time as you are in addiction treatment (13, collaborator experience)

Another aspect of offering treatment to a group of individuals suffering from a series of adverse experiences and social problems was that persons in treatment would often prefer to talk about those problems rather than the addiction problems, making the treatment difficult to manage for the counselors. During the interviews, the vast proportion of social problems was highlighted as leading to treatment adherence being unstable in addition to posing a risk of wearing the staff out. Several informants reported how both the social problems, addiction problems, and the linkage of these could not be solved by Allorfik alone but needed the involvement of several other sectors and areas: housing, education, Indigenous identity, and social services to name a few important ones, which are resources that were not always available. Several informants also pointed out that it was not the total amounts of substances used that constituted a problem but rather the pattern of substance use and/or binge drinking that seemed to be firmly rooted in parts of the society:

In other words, we drink fewer liters of alcohol per year, but when we do drink, we drink in the same way. (9, collaborator experience)

#### 3.4.3 No other place to go

Another aspect very much linked to the social burden among the people in treatment, and several interviewees highlighted how the treatment for the addiction transformed into a treatment for all the other painful things in life:

If it hadn't been for alcohol uhm addiction treatment, then they wouldn't have been treated at all for all the other sorrows or other pain they had in life, and that's what they got in Allorfik. That it is not only the abuse itself, but also all the pain that is included in that treatment. (14, staff experience)

The informants found that many of the persons in treatment struggled with social problems and some to a point where they could not stay in treatment and thus dropped out. Others would adhere to treatment but simultaneously struggle with social services in childcare cases resulting in staff from Allorfik taking it upon them to help these persons deal with social services. Moreover, some persons in treatment struggled with many difficulties except addiction problems, yet they still sought help in Allorfik, as they found nowhere else to go:

I see it a bit like there is simply not much help to be found anywhere. So just the fact that someone listens and wants to talk to them makes them happy. A place to go. You can't go for the municipality; you can't get hold of the municipality in any way… And we also had in Illulisat at one point, we were almost a crisis center too, if they were upset, they called and asked if they could come and talk. (6, both staff and collaborator experience)

Another interviewee described how Allorfik even sometimes was responsible for crisis management when someone was suicidal:

Yes, that, sometimes they send their citizens up here too um… I've had some like that, I've had a suicidal, a suicidal person. (12, staff experience)

## 4 Discussion

The overall finding of the present study was that while the process of implementation of the new national addiction treatment had been challenging, it was also considered successful by those involved. The informants found that it overall had led to making the intended services available to those in need. A key feature in the success of the implementation was stable management with a clear implementation plan according to the interviewees. Creating a shared language for treatment within Allorfik was an unforeseen challenge. These findings are not surprising. In general, a well-defined strategy for implementation is key for successful implementation as it provides the tactics for how to do it (35). Still, responsiveness and deliberate adaptations or modifications of elements during the implementation process are also considered valuable for a successful implementation (36). The latter may be especially important in an implementation such as Allorfik's as the establishment process was over a period of 3 years and the context of the implementation must be expected to be both complex and different in each location of implementation. When implementing Allorfik, adaptions were needed when faced with obstacles, for example, implementing new methodologies in treatment that revealed a need for a common language, continuous recruitment of (educated) staff, and building everything from the bottom over vast distances including a shared language in treatment while experiencing a high turnover in staff. A consistent management with a detailed understanding of the implementation strategy ensured that the plan was followed with the adaptations necessary (37).

The present study showed how the implementation of structures for collaborations with other sectors outside Allorfik had been only partly successful. Some collaborations were in effect, other collaborations were never established, and others had stopped because of changes in staff. The analysis illustrated that context and setting very much matter in the implementation (38, 39), and that may be particularly profound when implementing an intervention like Allorfik in a country like Greenland with vast distances and cultural differences on all levels. The healthcare system generally described the collaboration as good but seemed, in reality, to wish for an easy route to securely handing over persons to Allorfik rather than a wish for mutual collaboration. This wish may be an expression of reluctance in the health services towards addressing and getting involved in the treatment of patients' alcohol or cannabis use or a lack of resources to address these issues.

In the municipalities, there were (and still are) several different units within the organizations that were relevant for Allorfik to collaborate with, and while some collaborations were working fine, others were not. This difference seemed to be linked to resources or lack thereof in each place, thus pointing to the collaboration to have been very dependent on individuals rather than structures or procedures. This dependence on the individuals seemed to make the collaborations even more vulnerable with the high turnover in staff that was also described in the analysis. Informants from both internally in Allorfik and from collaborating partners were happy with the outreach work performed by Allorfik; however, this was also described as something that very much relied on the engagement of the individual counselors, which again made this work vulnerable to changes in staff. A high turnover in staff was also found to be a great challenge in the healthcare system (40) and a general problem across Greenland (41) and was thus not limited to Allorfik and probably not controllable by Allorfik either. The high turnover of staff combined with the descriptions of the multiple issues of the persons seeking treatment may pose a risk for staff burnout (42). This was mentioned by some interviewees framed as the job as a counselor might be too demanding for some persons.

The national plan recommended implementing MI and cognitive behavioral therapy as the basis of the new, public system but still continuing to offer the 12-step treatment through a private organization. Implementing new treatment methods presented a challenge to the implementation process since at the implementation start the available staff were originally trained in the 12-Step Model of treatment, and although the treatment context in Allorfik changed, it is well known that it can be difficult to change behavior and habit (43). The tensions generated by different views and experiences with treatment methodology also seemed to be a contributing factor to the high turnover in staff. Furthermore, the shifts in treatment models provided an unforeseen challenge as the known public narrative of good treatment referred to the 12-Step Model of treatment and the old treatment system. Allorfik thus had struggled to present a new success story to the public and change the narrative of treatment. Some interviewees pointed to this as a cause of people seeking treatment, however, a specific type of treatment, and others to how the narrative made the implementation more difficult, as these figures continued to put pressure on Allorfik.

Several informants considered Allorfik to be functioning better than the old treatment layout in various ways and emphasized that Allorfik has succeeded in offering treatment locally. However, the treatment-seeking persons' challenges turned out to be numerous, and it was considered that often the people approached Allorfik to seek help for their struggles in life in general rather than to seek treatment for their addiction problems. The large proportion of people in Greenland with not only problems with addictive behaviors but also suffering from having experienced abuse and neglect in Greenland is well established (1, 44–48). As pointed out by one interviewee, the levels of consumption of alcohol might have decreased over the past decades (49, 50), but the drinking pattern does not seem to have changed for the younger parts of the population (51). This is important to know because while Allorfik provided psychosocial treatment for the persons seeking help there, it would however be difficult for Allorfik to change societal structures that went beyond the persons entering the doors of the treatment centers.

The planning of the present study was inspired by the RE-AIM framework (28, 29), and although the purpose of this study was not to report on each of the main constructs in the framework, the study results however do reflect them. The present study found that the treatment reached both the intended population and beyond the intended population for treatment with the high turnup of people needing therapy. The interviewees reported that the new treatment was an improvement compared to the old treatment system, but this study cannot provide any further insight into the effectiveness of treatment and leaves this to be investigated in another study. Adoption seems to have been a struggle with the introduction of new methods and the turnover in staff; however, the interviewees report that the treatment service has only had a few adaptations, e.g., a guideline update. Implementation seems to have been a success and the treatment delivered seems to be in accordance with the treatment intended. The people entering treatment have an option of maintenance through the 6-month aftercare program, and in Allorfik, treatment maintenance quality also seemed to be a focus.

### 4.1 Strengths and limitations

The study has both strengths and limitations. We consider it a strength that the study was based on interviews with many different interviewees, representing different sectors and levels of the Greenlandic community, in addition to consisting of individuals with different experiences and involvement over a long period of time. It can be considered a limitation that almost all interviews were done in Danish, since this perhaps may have led some interviewees to feel insecure or withhold information due to language issues. However, all interviewees were allowed to do the interview in Greenlandic, and in all the Danish-speaking interviews, the interviewer emphasized the importance of speaking freely and using Greenlandic words or phrases if it was useful. All interviews also began with a turn of the table, where the interviewee was allowed to ask the interviewer questions of all kinds to try to make everyone more comfortable in the conversation and reverse the gaze (52).

It is a limitation that the study did not include interviewees from settlements and thus leaves out insight into the very remote local treatment and the people who travel great distances to treatment. This could perhaps have provided different perspectives to the study. However, the treatment services for the people living in Settlements had not changed considerably with the implementation of Allorfik as the new treatment centers were only in the five main cities.

It is a strength that the informants included inform the study with viewpoints that represent a broad picture from different areas and sectors, and not a small glimpse into time from a certain perspective which strengthens the study. The contributions of the reference group have also strengthened the study both when planning the study but also with the discussions around the emerging themes and interpretation of these, as the reference group both appreciated the aim of the study, contributed with ideas of improvement, and recognized the findings.

It may be considered a limitation that the study did not include any persons in or previously in treatment with Allorfik. This was a carefully considered choice that limited the perspective of this study. The study did not include persons in treatment firstly because the aim to investigate the process of implementation and organization did not correspond with the inclusion of treatment seekers as the treatment attendee's perspective would (hopefully) only provide a perspective of their own species treatment course during a few months and not the whole process of implementing Allorfik. If this issue were to be overcome, the study should have included persons who had attended treatment both before and after the implementation of Allorfik, which might be a particular group of individuals with particular, long-lasting problems. The study could also have included treatment seekers from the entire implementation period and preferably beyond to cover the process of implementation, which would have provided the study with an enormous data material. Lastly, the identification and recruitment of treatment seekers would provide the study with a completely different set of ethical considerations and a whole other perspective on the time and resources needed to complete the study. Thus, the inclusion of patients was avoided as it was deemed unfeasible for this study, but this does not mean that the perspective of the treatment seekers is not of importance. In contrast, the lack of treatment seekers’ perspective on Allorfik's organization may hopefully inspire a series of future studies.

### 4.2 Implications

With this study conducted and the implementation and organization outlined, the ground for continued work and future studies with the evaluation of Allorfik can then proceed. The findings suggest that the services in Allorfik were as intended and described, and thus there is ground for studying other essentials of Allorfik with treatment outcomes such as studies of treatment quality, register-based studies with treatment seekers, and treatment seekers’ perspectives of treatment services.

In Greenland, evidence of Allorfik’s implementation and organization will be beneficial to both policymakers funding Allorfik and internally in Allorfik, where the findings can be useful in organizational development work. The findings of this study support the possibility of successfully implementing a new treatment service over vast distances and point to some of the elements aiding the success, which could be useful both internally in Greenland for other services and in other Indigenous communities with similar challenges.

## 5 Conclusion

In conclusion, the implementation and organization of Allorfik seem to have been delivered as intended and promised according to those involved. The process of implementation has been challenging, and adaptations of the original plan have been made to reach a well-functioning service, but overall, the organization and treatment services intended were the organization and treatment implemented. However, Allorfik also seems to have become more than the addiction treatment service it was planned to be—it has developed into a service with easy access for the citizens in need of help who are otherwise limited by waiting in phone lines, for a referral or the next specialist visit, or simply not available due to limited resources in Greenland. Allorfik had on top of the addiction treatment services developed into a crisis management center for some and a safe space for others to talk about their past and present struggles in life, all while still providing treatment for addiction problems for the people in need.

## Data Availability

Data management and storage were handled by the Open Patient Data Explorative Network. The datasets presented in this article are not readily available because the interview data is in the Danish language only. Requests to access the datasets should be directed to jholflod@health.sdu.dk.

## Footnotes

^1^The new guideline was a simplified instruction for the counselors, which aimed to provide the tools for a better guidance through the treatment course for counselors and the people in treatment.

^2^A municipality health prevention worker would typically conduct local prevention and health promotion initiatives and projects, arrange thematic courses, and collaborate with all relevant institutions in the local area.

## Supplementary material

Supplementary Appendix I Table 1, Supplementary Appendix II The interviewees, their time of involvement with Allorfik and their position as developer/stakeholder. Informed consent for study participants.
