# Supplementary material for: A qualitative study of the implementation and organization of the national Greenlandic addiction treatment service
Source: Front Health Serv. 2024 Mar 6;4:1219787. doi: 10.3389/frhs.2024.1219787 (PMC10950955; doi:10.3389/frhs.2024.1219787)
Supplement: Supplementary Appendix I Table 1, Supplementary Appendix II — The interviewees, their time of involvement with Allorfik and their position as developer/stakeholder. Informed consent for study participants. [file Datasheet1.zip › Data Sheet 1_v1/Appendix II.DOCX]

**Samtykkeerklæring til behandling af sensitive data til forskningsprojekt**

I forbindelse med din deltagelse i et forskningsprojekt på Ilisimatusarfik og Syddansk Universitet, har vi brug for dit samtykke til, at vi må behandle dine oplysninger fra et interview.

Til projektet: *Implementation of best practice models in treatment of addiction of alcohol and other drugs in Greenland,* gennemføres interviews om Allorfik, som vil blive optaget (på video/lydfil), transskriberet og analyseret. Data herfra vil indgå i det samlede studie og forventes også publiceret i en videnskabelig artikel som et delstudie. Analysen vil blive diskuteret med projektets referencegruppe, som bidrager til at kvalificere indholdet og sikre at alle særlige etiske forhold for små populationer er imødekommet.

Samtykket er **frivilligt**, og du kan **til enhver tid trække dit samtykke til behandlingen af personoplysninger tilbage** ved at kontakte: Julie Flyger Holflod på mail: [jholflod@health.sdu.dk](mailto:jholflod@health.sdu.dk) eller tlf.: +45 2537 6425

**Samtykkeerklæring til brug og opbevaring af de oplysninger fra interview**

Tak fordi du har valgt at deltage som interviewperson i forskningsprojektet omkring evaluering af Allorfiks rusmiddelbehandling.

Projektet hedder på engelsk: *Implementation of best practice models in treatment of addiction of alcohol and other drugs in Greenland* og foregår på Ilisimatusarfik og Syddansk Universitet.

I forbindelse med din deltagelse har vi brug for, at du giver dit samtykke til at vi må bruge de oplysninger, der kom frem under interviewet i det videre arbejde med projektet.

l projektet gennemføres interviews, som vil blive optaget (på video/lydfil), transskriberet og analyseret. Data herfra vil indgå i det samlede studie og forventes publiceret i en videnskabelig artikel på engelsk som en del af den samlede undersøgelse.

I analysen fremgår interviewpersoner ikke med navn. Analysen vil også blive diskuteret med projektets referencegruppe, som bidrager til at kvalificere indholdet og sikre at alle særlige etiske forhold for små populationer er imødekommet.

Samtykket er **frivilligt**, og du kan **til enhver tid trække dit samtykke til brug af data fra interviewet tilbage** ved at kontakte: Julie Flyger Holflod på mail: [jholflod@health.sdu.dk](mailto:jholflod@health.sdu.dk) eller tlf.: +45 2537 6425

**Declaration of consent for the processing of sensitive data for a research project**

In connection with your participation in a research project at Ilisimatusarfik and the University of Southern Denmark, we need your consent to process your information from an interview.

For the project: Implementation of best practice models in treatment of addiction of alcohol and other drugs in Greenland, interviews will be conducted about Allorfik, which will be recorded (on video/audio file), transcribed and analysed. Data from this will be included in the overall study and is also expected to be published in a scientific article as a sub-study. The analysis will be discussed with the project's reference group, which contributes to qualifying the content and ensuring that all special ethical conditions for small populations are met.

Consent is voluntary, and you can withdraw your consent to the processing of personal data at any time by contacting: Julie Flyger Holflod by email: jholflod@health.sdu.dk or phone: +45 2537 6425

**Declaration of consent for the use and storage of the information from the interview**

Thank you for choosing to participate as an interviewee in the research project regarding the evaluation of Allorfik's drug treatment.

The project is called in English: Implementation of best practice models in treatment of addiction of alcohol and other drugs in Greenland and takes place at Ilisimatusarfik and the University of Southern Denmark.

In connection with your participation, we need you to give your consent for us to use the information that came out during the interview in the further work with the project.

In the project, interviews will be conducted, which will be recorded (on video/audio file), transcribed and analysed. Data from this will form part of the overall study and is expected to be published in a scientific article in English as part of the overall study.

In the analysis, interviewees do not appear by name. The analysis will be discussed with the project's reference group, which contributes to qualifying the content and ensuring that all special ethical conditions for small populations are met.

Consent is voluntary, and you can withdraw your consent to the use of data from the interview at any time by contacting: Julie Flyger Holflod by email: jholflod@health.sdu.dk or tel.: +45 2537 6425

​
